# Functional Ecology of Two Contrasting Freshwater Ciliated Protists in Relation to Temperature

**DOI:** 10.1111/jeu.12823

**Published:** 2020-12-27

**Authors:** Xiaoteng Lu, Yunyi Gao, Thomas Weisse

**Affiliations:** ^1^ Research Department for Limnology Mondsee, University of Innsbruck Mondseestrasse 9 Mondsee A‐5310 Austria; ^2^ Institute of Evolution & Marine Biodiversity Ocean University of China Qingdao 266003 China

**Keywords:** Bottom‐up control, freshwater ciliates, functional response, numerical response, phylogeny, temperature response

## Abstract

We conducted microcosm experiments with two contrasting freshwater ciliates on functional traits (FTs) related to their growth rate (numerical response, NR) and ingestion rate (functional response, FR) over a range of ecologically relevant temperatures. *Histiobalantium bodamicum* and *Vorticella natans* are common planktonic ciliates but their abundance, swimming behavior, and temperature tolerance are different. In contrast to most sessile peritrich species, the motile *V. natans* is not strictly bacterivorous but also voraciously feeds upon small algae. We observed three main alterations in the shape of NR of both species with temperature, that is, change in the maximum growth rate, in the initial slope and in the threshold food level needed to sustain the population. Similarly, maximum ingestion rate, gross growth efficiency (GGE), and cell size varied with temperature and species. These findings caution against generalizing ciliate performance in relation to the ongoing global warming. Our results suggest that *V. natans* is the superior competitor to *H. bodamicum* in terms of temperature tolerance and bottom‐up control. However, the abundance of *V. natans* is usually low compared to *H. bodamicum* and other common freshwater ciliates, suggesting that *V. natans* is more strongly top‐down controlled via predation than *H. bodamicum*. The taxonomic position of *V. natans* has been debated. Therefore, to confirm species and genus affiliation of our study objects, we sequenced their small subunit ribosomal RNA (SSU rDNA) gene.

IT has been known for decades that protists represent central nodes in aquatic food webs (Fenchel 1987). As primary producers, predators, food, and parasites, they are structural elements of any aquatic food web and are of tremendous global and local significance for cycling of matter in the ocean and inland water bodies (Azam et al. 1983; Fenchel 1992; Porter et al. 1985; Sherr and Sherr 1994; Weisse 2003; Weisse et al. 2002). Protists are ideal candidates for testing general ecological and evolutionary principles, because they are easy to cultivate in large cell numbers, have short generation times, and can be manipulated with ease (Weisse et al. 2016). The ciliated protozoa (phylum Ciliophora) are generally considered the most evolved and complex organisms among the protists (Hausmann and Bradbury 1996; Lynn 2008). However, compared to macroorganisms and other microorganisms such as bacteria and algae, ciliates received little attention in trait‐based functional ecology thus far (Fenchel 1987; Weisse 2017; Weisse et al. 2016).

Ciliates are primarily bottom‐up controlled via resources (food) interacting with abiotic factors (temperature, salinity, pH) in most natural environments (Calbet and Saiz 2005; Galbraith and Burns 2010; Weisse 2006). Nevertheless, the nature of this interaction has been studied and quantified only with a few selected species (Montagnes 2013; Verity 1985; Weisse and Stadler 2006; Weisse et al. 2002). Therefore, it is at present not possible to accurately predict population growth rates of ciliates in situ from laboratory‐derived maximum growth rates (Gaedke and Straile 1994; Macek et al. 1996; Montagnes 1996; Šimek et al. 2000).

Bottom‐up control can be measured by numerical response (NR) and functional response (FR) of the predator in relation to food concentration and abiotic parameters. The NR characterizes the growth response of a predator in relation to prey availability; FR measures prey ingestion as a function of food biomass (reviewed by Montagnes 2013 and Weisse et al. 2016). Using nonlinear curve fitting analogous to Michaelis–Menten enzyme kinetics and to the relationship between photosynthesis and irradiance (P‐I curve) of algae, NR and FR of heterotrophs can be parameterized, yielding several characteristic parameters. In NR, the maximum specific growth rate (µ_max_), which is reached at satiating prey levels, denotes the potential of an organism to increase its population size under optimum conditions. The initial slope (α) of the NR curve characterizes the affinity between prey and predator, and the threshold prey concentration (*P*′), at which population growth equals mortality, determines predator survival at low food levels. The maximum ingestion rate (*I*_max_) and the half‐saturation constant (*k*) of the FR curve indicate the grazing pressure of a predator on its prey at high and moderate food levels. These ecophysiological parameters represent functional response and effect traits (reviewed by Weisse 2017) that can be used to assess the competitive advantages of zooplankton (Lampert 1977; Montagnes 1996).

Temperature, as the most important abiotic variable to affect the physiological rates of all ectotherms, represents the main physical dimension of bottom‐up control of ciliate population dynamics in freshwater (Krenek et al. 2011; Montagnes et al. 2008; Weisse and Stadler 2006). The upper temperature tolerance limit (UTTL) of many aquatic species including ciliates is generally related to the habitat temperature (Gächter and Weisse 2006; Weisse et al. 2001; Weisse and Montagnes 1998) but may be above the maximum temperature encountered in situ (Krenek et al. 2011; Martinez 1980). This "thermal safety margin” should allow many species to cope with global warming (Krenek et al. 2012). However, in the course of climate change, the UTTL of temperature‐sensitive protist species may constrain their distribution and lead to a shift in the species composition (Montagnes et al. 2008; Wirth et al. 2019).

Thus far, only few NR and FR data are available for freshwater ciliates, and the temperature effect on NR and FR has been studied with even less planktonic ciliates (Weisse 2017; Weisse et al. 2002 and references therein). To this end, we used two contrasting planktonic ciliates, *Histiobalantium bodamicum* (Fig. 1A–C) and *Vorticella natans* (Fig. 1G–J), to examine the combined effect of food concentration and temperature on growth, ingestion, and cellular biomass. *Vorticella natans* and *H. bodamicum* are common planktonic species, often co‐occur and are similar in size (Foissner et al. 1999). However, their abundance and swimming behavior are clearly different: *Histiobalantium bodamicum* is a fast moving species that may reach high abundances; maxima of 34,500 cells/liter were reported from L. Zurich (Posch et al. 2015), 6,400 cells/liter were reported from L. Constance (Müller and Weisse 1994), and 3,800 cells/liter were reported from L. Mondsee (Nachbaur 2017). *Vorticella natans*, on the other hand, a nonsessile peritrich ciliate, moves slowly, but is rarely abundant. For instance, 24–40 cells/liter have been recorded in a betamesosaprobic reservoir (Foissner et al. 1999) and 14–223 cells/liter in oligo‐mesotrophic Lake Mondsee (Nachbaur 2017).

**Figure 1:**
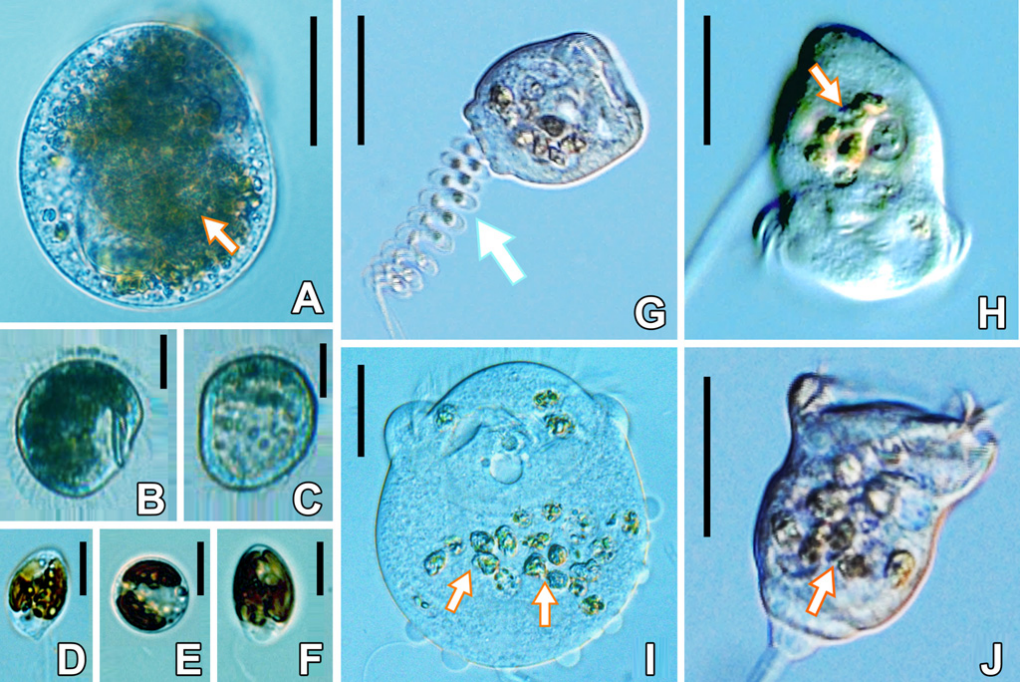
Live observations of *Histiobalantium bodamicum* (**A–C**), *Vorticella natans* (**G–J**), and the prey *Cryptomonas* sp. strain 26.80 (**D–F**). Arrows in (A) and (H–J) show the ingested *Cryptomonas* sp. strain 26.80; the arrow in (G) marks the helical contracting stalk; (B) and (C) are FlowCAM images captured at 18 and 18.5 °C, respectively. Note that when temperature increased above 18 °C, *H. bodamicum* turned from a fit feeding state (B) to a starved state (C). Scale bar = 30 µm in (A); = 20 µm in (B, C); = 4 µm in (D–E); 25 µm in (G, H, J); = 10 µm in (I).

This is the first study investigating functional traits of *V. natans*. For *H. bodamicum*, earlier work investigated its NR of FR at 15 °C (Müller and Schlegel 1999; Müller and Weisse 1994). We complemented these studies by investigating the NR and FR of this ciliate over temperatures ranging from 5 to 20 °C. We hypothesized that the rare species, *V. natans*, is the inferior competitor, relative to the abundant species *H. bodamicum*, in terms of bottom‐up control at low‐to‐moderate temperatures (H_1_).

This work is part of our endeavor to apply protist microcosms to investigate general concepts in functional ecology such as the relation between food and reproduction (i.e. specific growth rate in protists) and the temperature effect on physiological key variables. We use NR and FR data on freshwater ciliates to advance their trait‐based functional classification in relation to temperature. Following the independent response model (Fenton et al. 2010; Li and Montagnes 2015; Weisse et al. 2016), we did not assume a direct link between the ciliates’ NR and FR, mediated by a constant conversion efficiency (or assimilation efficiency, gross growth efficiency). These efficiencies are associated with each other but not identical, reviewed by Weisse et al. (2016). For pragmatic reasons, we calculated gross growth efficiency (GGE), which has been compiled for a number of marine and freshwater ciliates (reviewed by Hansen et al. 1997; Straile 1997). We hypothesized that GGE would vary with temperature (H_2_), as it has been demonstrated earlier for several ciliate and dinoflagellate species (Fenton et al. 2010; Li and Montagnes 2015; Straile 1997; Weisse 2004). Our results demonstrate that temperature affects all parameters of the NR and FR curves and GGE of ciliates species specifically. Importantly, the temperature effect did not always follow general trends derived from earlier studies and theoretical considerations. Therefore, results of this study caution against generalizing the role of ciliates in aquatic food webs in relation to the ongoing global warming.

## Materials and Methods

### Study organisms

The hymenostomatid ciliate *H. bodamicum* (Fig. 1A–C; size in vivo ca. 40–60 × 35–45 μm, average cell volume ~ 32,000 µm^3^) and the free‐living peritrich ciliate *V. natans* (Fig. 1G–J; live cell size 30–50 × 30–50 μm, average cell volume ~ 20,000 µm^3^; Table 1) were isolated by enriching natural samples from oligo‐mesotrophic Lake Mondsee, Austria, with *Cryptomonas* sp. strain 26.80 (Fig. 1D–F) as food. Clonal cultures of both ciliate species were established by a minimum of three serial, single cell isolations, using sterile‐filtered water from Mondsee to avoid contamination with other cells. Images of live *H. bodamicum* and *V. natans* cells were taken by FlowCAM (Flow Cytometer And Microscope FlowCam^®^; Fluid Imaging Technologies, Inc., Yarmouth, ME) at 5–20 °C and exported to Nikon imaging analysis (NIS‐Elements D 3.2 64‐bit) to more accurately size the images to the nearest 0.1 µm. Cell volumes were calculated from length and width measurements assuming the shape of a flattened ellipsoid for *H. bodamicum* (www.planktonforum.eu) and a prolate ellipsoid for *V. natan*s. We converted cell volume (in μm^3^) to cell biomass (ng C/cell) assuming C = 0.216 × volume^0.939^ (Menden‐Deuer and Lessard 2000) (Table 1).

**Table 1:** Cell volume and cell biomass of *Histiobalantium bodamicum* and *Vorticella natans* at moderate food level (0.5 mg C/liter) and temperatures ranging from 5 to 20 °C

Temperature (°C)	Species	Cell volume (µm^3^)	Cell biomass (ng C/cell)
5	*H. bodamicum*	40,941 ± 9,722	4.6 ± 1.0
	*V. natans*	20,539 ± 9,567	2.4 ± 1.1
10	*H. bodamicum*	34,628 ± 8,581	3.9 ± 0.9
	*V. natans*	22,068 ± 8,932	2.6 ± 1.0
15	*H. bodamicum*	26,749 ± 7,471	3.1 ± 0.8
	*V. natans*	29,455 ± 10,153	3.4 ± 1.1
18	*H. bodamicum*	32,557 ± 8,795	3.7 ± 0.9
20	*V. natans*	17,848 ± 7,938	2.1 ± 0.9

### Food organism

*Cryptomonas* sp. strain 26.80 (Fig. 1D–F) was used as food source, provided by the Culture Collection of Algae in Göttingen (Germany). Its cell size was measured by an electronic particle analyzer (CASY 1‐model TTC; Schärfe System, Reutlingen, Germany) at ~15 × 10^6^ cells/liter, corresponding to approximately 0.5 mg C/liter. The measuring principle of the particle analyzer was described by Weisse and Kirchhoff (1997). Average algal cell volume over the temperature range investigated was 293 ± 37 μm^3^. We converted cell volume (in μm^3^) to carbon units (C, in pg/cell) assuming C = 0.261 × volume^0.860^ (Menden‐Deuer and Lessard 2000) (Table 2). Note that the carbon conversion factors used for ciliates and algae are slightly different, depending on their different cell size (table 4 in Menden‐Deuer and Lessard 2000). This *Cryptomonas* strain has been used as standard food in many previous experiments with various planktonic ciliates (Müller and Geller 1993; Müller and Schlegel 1999; Weisse et al. 2002).

**Table 2:** Cell volume and cell biomass of *Cryptomonas* sp. strain 26.80 at temperatures ranging from 5 to 20 °C

Temperature (°C)	Diameter (µm)	Cell volume (µm^3^)	Biomass (pg C/cell)
5	8.5	337	39
10	8.2	304	36
15	8.3	313	37
18	7.8	262	31
20	7.6	247	30
Average for all	8.1	293	34
Standard deviation	0.4	37	4

Ciliates were maintained with *Cryptomonas* sp. in sterile‐filtered Mondsee water. The prey was incubated in pure MWC medium. Ciliate clonal cultures were not axenic, but in our exponentially growing cultures and in the experiments, bacterial biomass was mostly minor, relative to that of *Cryptomonas* (Table S1), and both ciliates readily ingested the algae (Fig. 1). Previous studies demonstrated that *H. bodamicum* does not feed extensively on bacteria if suitable flagellates are abundant (Foissner et al. 1999; Müller and Schlegel 1999; Müller and Weisse 1994). For all experiments, both ciliates and prey cultures were harvested in their exponential phase.

### Experimental design

Ciliates were taken from stock cultures maintained at 15 °C under continuous light (100 μmol photons/m^2^/s). An inoculum (20 ml) was transferred to 250‐ml tissue culture bottles, containing 180 ml of sterile‐filtered Mondsee water; to obtain the exponential growth of ciliates, 1–2 ml from the target prey *Cryptomonas* sp. stock culture was added. Accordingly, the MWC medium present in the algal cultures was highly diluted (dilution factor 100–200 fold), and the experimental wells (see below) contained only traces of the MWC medium. Experiments were performed over temperatures ranging from 5 to 20 °C at 5 °C intervals. However, *H. bodamicum* did not grow at 20 °C. Therefore, we conducted an additional experiment at 18 °C for this species. The target temperatures were reached by changing the incubation temperature by 1 °C per d; that is, both the ciliates and their prey were stepwise acclimated to the experimental temperatures over a period of 5 d.

The light level for ciliates and prey during the acclimation period was ~70 μmol photons/m^2^/s. Ciliate abundance and fitness were examined by live observation under a dissecting microscope daily; prey abundance was monitored with the electronic particle counter and adjusted to the target density if necessary. Experiments were conducted in 24‐well tissue culture test plates (TPP cat. no. 92024), and each well represented a single growth treatment. Forty ciliate cells taken from the tissue culture bottles were carefully pipetted into each well, containing 2 ml of sterile‐filtered Mondsee water with acclimated prey. Thus, the initial ciliate concentration in each well was 20 cells/ml. We used 24 prey concentrations in a modified geometric progression method, increasing prey abundance by a factor of 1.1–1.5 between each neighboring well, beginning with the lowest prey level. The prey level ranged from 1 × 10^3^ to 1 × 10^5^ cells/ml, corresponding to carbon levels from 0.02 to 7.0 mg C/liter. This method yields more accurate parameter estimates for curve fitting than replicating standard food levels (Montagnes and Berges 2004). Controls for prey growth, without ciliates, were run at identical food concentrations at each temperature.

To account for the contribution of bacteria to total initial prey biomass, we measured bacterial abundance and cell size at 15 °C, using epifluorescence microscopy after staining with DAPI (Porter and Feig 1980) at low (1,000 cells/ml), medium (15,000 cells/ml), and high (100,000 cells/ml) *Cryptomonas* food levels (Table S1). The mean bacterial size was 0.65 µm in diameter, 1.8 μm in length, and 0.60 μm^3^ in volume assuming the geometrical shape of a rod. We converted bacterial cell volume (in μm^3^) to cell biomass (fg C/cell) assuming C = 133.754 × volume^0.438^ (Romanova and Sazhin 2010), yielding 106.7 fg C/cell.

Prior to the beginning of the experiments, ciliates and controls were acclimated to the experimental conditions in the wells for 24 h at each temperature and food concentration. After 24 h, the experimental incubation began and lasted for 48 h. Light levels were identical to those used during the acclimation period.

Subsamples (1 ml each) were taken from each well at the beginning and end of each experiment (*t*_0_ and *t*_2_) and fixed with acid Lugol’s iodine (final concentration 2%, vol/vol) for microscopic analyses. Preliminary experiments with both ciliate species had shown that ciliate growth rates were not significantly affected if the experimental volume was reduced from 2 to 1 ml (X. Lu, unpubl. obs.). Ciliates and prey levels were measured by counting cells in a Sedgewick‐Rafter counting chamber (1 ml vol.). In the initial 1 ml subsamples, approximately 20 ciliates were counted each; in the final subsamples, the ciliate density was more variable, ranging from 10 to 50 cells/ml. Since we counted the ciliates in 1 ml subsamples, this is identical to the number of ciliates counted. Depending on the algal density, cells were counted in 5–20 individual squares (corresponding to 1 µl each) or several rows (corresponding to 50 µl each) of the Sedgewick‐rafter cell. The total algal cell counts in the subsamples ranged from ~ 200 to 600 cells.

### Calculation of experimental results

Ciliate growth rates were determined from end‐point measurements of cell number, assuming exponential growth over the experimental period according to Equation (1):

(1) $$\mu =\ln\;(N_{t}/N_{0})/t$$

*N*_0_ and *N_t_* denote ciliate numbers at the beginning and end of the experiment, respectively; μ (d^−1^) is the specific growth rate, and *t* is the duration of the experiment (d).

Ciliate growth rates were related to the geometric mean prey concentration (*P*) during the experimental period (Frost 1972; Heinbokel 1978). The latter was calculated according to Equation (2):

(2) $$P=\left(P_{t}‐P_{0}\right)/\text{ln(}P_{t}‐P_{0})$$

where *P*_0_ and *P_t_* are the initial and final prey concentrations (ng C/ml).

Numerical response data were fit to Equation (3), which includes a positive *x*‐axis intercept, using the Marquardt–Levenberg algorithm (SigmaPlot, version 14):

(3) $$\mu =\mu_{\max}\left(P‐P^{\prime }\right)/\left(k_{2}+P‐P^{\prime }\right)$$

μ_max_ is the maximum specific growth rate (d^−1^), *P* is the mean prey concentration (Equation 2), *k*_2_ is a constant (mg C/liter), and *P*′ is the *x*‐axis intercept (i.e. the threshold concentration, where μ = 0). This equation is analogous to the Michaelis–Menten model and Holling’s type II functional response (Holling 1959), but assumes a positive *x*‐axis intercept where population growth equals mortality (reviewed by Montagnes 2013; Weisse et al. 2016). Note that the constant *k_2_* of Equation (3) is not identical to the half‐saturation constant known from Michaelis–Menten kinetics.

Ciliate ingestion rate (*I*, ng C/Cil/d) was calculated according to

(4) I=(P×g)×m/Rm

where *g* is the grazing rate (d^−1^), *m* is the cellular biomass of *Cryptomonas* sp. (ng C/cell), and *Rm* is the mean ciliate abundance (per ml) in the experimental containers. Grazing rate (*g*) was calculated as

(5) $$g=\left(\text{ln (}C_{t}/C_{0})‐\text{ln (}P_{t}/P_{0})\right)/t$$

where *C*_0_ and *C_t_* are the initial and final *Cryptomonas* sp. numbers in the controls; *g* is equivalent to μ of *Cryptomonas* sp. observed in the controls minus growth rates measured in the experimental containers. We attempted to fit the functional response (FR) of both species to Holling’s type II curvilinear model (Holling 1959):

(6) $$I=I_{\max}\left(P\right)/\left(k+P\right)$$

where *I*_max_ (ng C/Cil/d) is the maximum ingestion rate and *k is* half‐saturation constant (mg C/liter), indicating prey concentration that results in 0.5 × *I*_max_. However, at high temperatures, ingestion rates of both species fitted best to a linear model.

Gross growth efficiency (GGE) is the fraction of prey biomass converted into predator biomass:

(7) GGE=μ×M/I

where µ (d^−1^) is the growth rate, *M* is the cellular biomass of the ciliates (ng C/ciliate), and *I* is the specific ingestion rate (ng C/cil/d).

### DNA extraction, PCR amplification, and sequencing

Five cells were isolated from each ciliate culture and washed 3–5 times with sterile‐filtered (0.22 µm) Mondsee water to remove potential contaminants. These cells were then separated into three groups, with one (two replicates) and three cells each. Each of the groups was subsequently transferred to a 1.5‐ml microfuge tube with a minimum volume of water and sequenced separately. Genomic DNA was extracted separately from these three groups, using the DNeasy Blood and Tissue Kit (QIAGEN, Hilden, Germany) according to the protocol of manufactures. The small subunit ribosomal RNA gene (SSU rDNA) was amplified using Q5^®^ Hot Start High‐Fidelity DNA Polymerase (New England BioLabs, Frankfurt am Main, Germany) with primers of 82F (5′‐GAA ACT GCG AAT GGC TC‐3′) and 18sR (5′‐GAT CCT TCT GCA GGT TCA CCT AC‐3′) (Elwood et al. 1985; Medlin et al. 1988). The touchdown PCR program was designed with the annealing temperature of 69–51 °C (Wang et al. 2017). The PCR products were sequenced bidirectionally by TSINGKE Incorporated Company (Qingdao, China). The contigs were assembled by SeqMan (DNAStar).

### Phylogenetic analyses

Using the online program GUIDANCE2 Server (http://guidance.tau.ac.il/ver2/) with default settings (Sela et al. 2015), the newly characterized SSU rDNA sequences of *H. bodamicum* and *V. natans* were aligned with 134 other ciliate sequences obtained from NCBI GenBank database. The accession numbers are shown after the species names in the phylogenetic tree. *Bryometopus atypicus* (EU039886), *Platyophrya vorax* (AF060454), and *Colpoda inflata* (M97908) were selected as out‐group taxa. These sequences were edited manually using BioEdit 7.2.5 in order to remove ambiguous gaps (Hall 1999). Maximum‐likelihood (ML) analysis was performed in CIPRES Science Gateway (http://www.phylo.org/) using RAxML‐HPC2 on XSEDE v8.2.10 (Stamatakis et al. 2008) with the model of GTR + I + G selected by MrModeltest v2.0 (Nylander 2004). Bayesian inference (BI) tree was constructed using MrBayes on XSEDE v3.2.6 in CIPRES Science Gateway with the model of GTR + I + G selected by MrModeltest v2.0 (Nylander 2004). MEGA v6.06 (Tamura et al. 2013) was used to visualize tree topologies.

## Results

### Numerical response—functional traits related to temperature

We wanted to assess NR of both ciliates over temperatures ranging from 5 to 20 °C. However, cell numbers of *H. bodamicum* rapidly declined at 20 °C. Our repeated, failed attempts to rear this ciliate at 19–20 °C suggest that the UTTL of this species is close to 18.5 °C. Therefore, we report results at 18 °C, the highest temperature at which positive growth was obtained for this species over several days.

**Figure 2:**
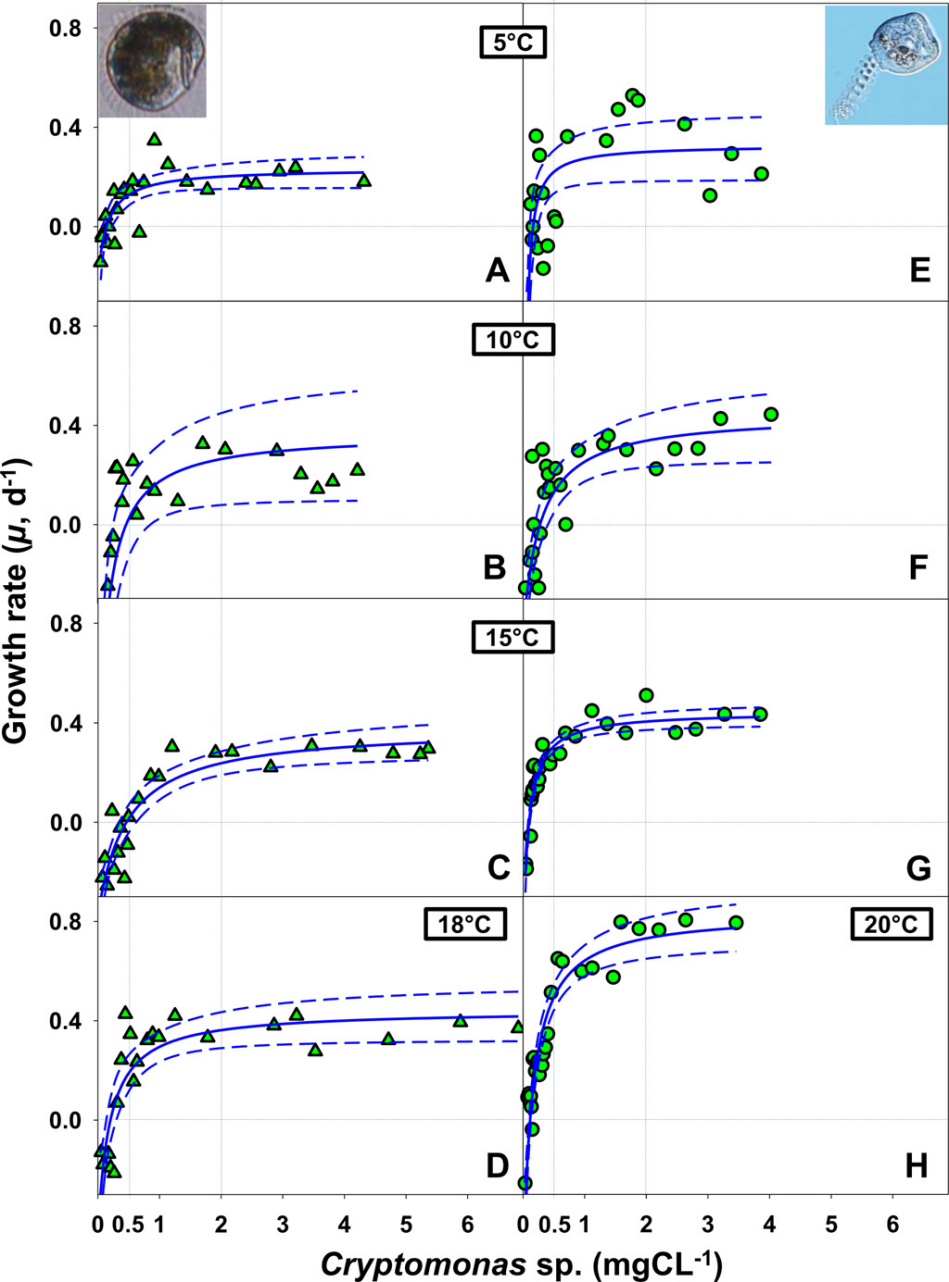
Numerical response of *Histiobalantium bodamicum* (**A–D**) and *Vorticella natans* (**E–H**) over temperature ranging from 5 to 20 °C and food levels ranging from 0.02 to 7.0 mg C/liter. Horizontal dotted lines mark the zero growth rate; vertical lines show moderate (0.5 mg C/liter) and high (2.0 mg C/liter) food concentrations. Dashed lines mark the 95% confidence interval of the curves.

**Figure 3:**
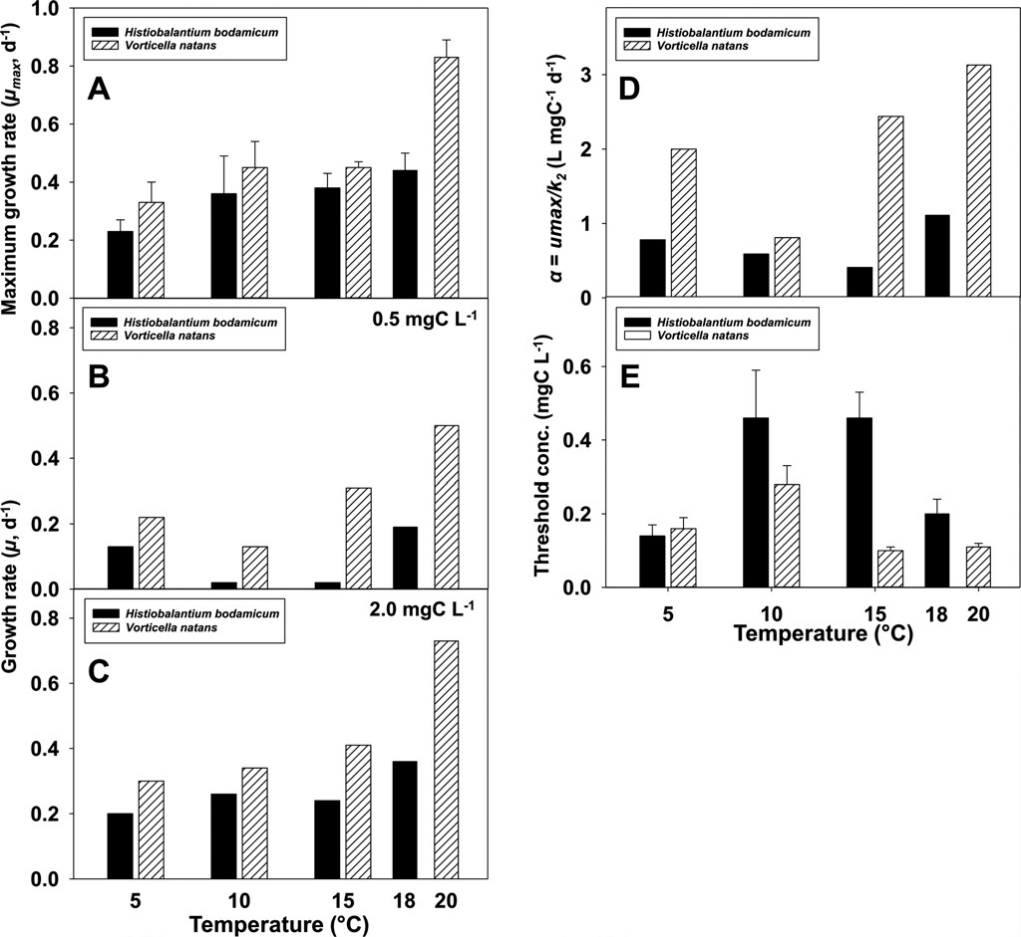
Temperature response (5–20 °C) of *Histiobalantium bodamicum* and *Vorticella natans* of five parameters derived from their numerical responses. (**A**) Maximum growth rate (µ_max_) vs. temperature; (**B, C**) growth rate at 0.5 and 2.0 mg C/liter, respectively; (**D**) relationship between the initial slope (α) of the numerical response vs. temperature; (**E**) the relationship between the threshold level (where growth rate is zero) vs. temperature. Error bars represent standard errors.

At all temperatures tested, growth rate of *H. bodamicum* (Fig. 2A–D) and *V. natans* (Fig. 2E–H) followed a rectangular hyperbolic response to food concentration. Curve fitting (Equation 3) yielded significant parameter estimates for µ_max_, *P*′ and *k*_2_ (Table 3). In both species, µ_max_ was positively related to temperature, that is, µ_max_ of *H. bodamicum* (0.44 d^−1^) peaked at 18 °C and µ_max_ of *V. natans* (0.83 d^−1^) at 20 °C (Fig. 3A and Table 3). At moderate temperatures (10–15 °C), µ_max_ was constant for both species, that is ~0.37 d^−1^ for *H. bodamicum* and 0.45 d^−1^ for *V. natans*. Lowest µ_max_, 0.23 d^−1^ for *H. bodamicum* and 0.33 d^−1^ for *V. natans* were recorded for both species at the lowest temperature tested (5 °C). However, the parameter µ_max_ may not provide a realistic estimate of maximum specific growth if an asymptote is not reached at meaningful food concentrations; that is, in some cases (Fig. 2B, F, H), µ_max_ predicted by the curve fitting (Equation 3) was reached at very high food levels (> 6 mg C/liter) that are rarely met in the natural environment. Therefore, we compared growth rates, predicted by Equation (3), for each temperature at 0.5 and 2.0 mg C/liter, respectively (Fig. 3B, C and Table 3). These two carbon levels represent moderate and high food concentrations, corresponding to peak levels in oligo‐mesotrophic (0.5 mg C/liter) lakes and typical concentrations found in many eutrophic lakes (2.0 mg C/liter) (De Kluijver et al. 2014; Weisse et al. 2002). Due to bacterial contamination, the actual food levels may have been slightly higher (Table S1, see Discussion).

**Table 3:** Parameter values (±SE) of numerical responses referring to the curves presented in Fig. 2 and 3

Temp. (°C)	Species	Parameters						
		μ_max_ (d^−1^) ± SE	*P*‐value_μ_max_	*P*′ (mg C/ml) ± SE	*P*‐value_*P*′	*k*_2_ (mg C/ml) ± SE	*P*‐value_*k*_2_	α = μ_max_/*k*_2_ (per mg C/d ml)
5	*Histiobalantium bodamicum*	0.23 ± 0.04	< 0.0001	0.14 ± 0.03	0.0002	0.30 ± 0.14	0.0421	0.78
	*Vorticella natans*	0.33 ± 0.07	< 0.0001	0.16 ± 0.03	< 0.0001	0.16 ± 0.06	0.0114	2
10	*H. bodamicum*	0.36 ± 0.13	0.0086	0.46 ± 0.13	0.0014	0.61 ± 0.20	0.0054	0.59
	*V. natans*	0.45 ± 0.09	< 0.0001	0.28 ± 0.05	< 0.0001	0.55 ± 0.19	0.0078	0.81
15	*H. bodamicum*	0.38 ± 0.05	< 0.0001	0.46 ± 0.07	< 0.0001	0.93 ± 0.22	0.0003	0.41
	*V. natans*	0.45 ± 0.02	< 0.0001	0.10 ± 0.01	< 0.0001	0.18 ± 0.02	< 0.0001	2.44
18	*H. bodamicum*	0.44 ± 0.06	< 0.0001	0.20 ± 0.04	< 0.0001	0.40 ± 0.12	0.0045	1.11
20	*V. natans*	0.83 ± 0.06	< 0.0001	0.11 ± 0.01	< 0.0001	0.27 ± 0.04	< 0.0001	3.13

Irrespective of temperature and food level, *V. natans* reached higher growth rates than *H. bodamicum* in each case (Fig. 3B, C).

The initial slope (α = µ_max_/*k*), that is, the affinity between the ciliate and its prey, of *V. natans* was also higher than that of *H. bodamicum* at each temperature (Fig. 3D). In both species, α peaked at the highest temperature tested.

The threshold food concentration (*P*′) of *H. bodamicum* was higher than that of *V. natans* at all temperatures tested except at 5 °C (Fig. 3E). The temperature response of *P*′ was unimodal in both species, peaking at 10–15 °C for *H. bodamicum* (0.46 mg C/liter), respectively, at 10 °C for *V. natans* (0.28 mg C/liter). At the upper temperature tolerance limit of *H. bodamicum* (18 °C), its *P*′ was 0.20 mg C/liter, that is, almost twice as high as that measured for *V. natans* at 20 °C (0.11 mg C/liter). At 5 °C, *P*′ calculated for both species (0.14–0.16 mg C/liter) were not different, because their standard errors (Fig. 3E) and prediction intervals (not shown) overlapped.

### Functional response in relation to temperature

**Figure 4:**
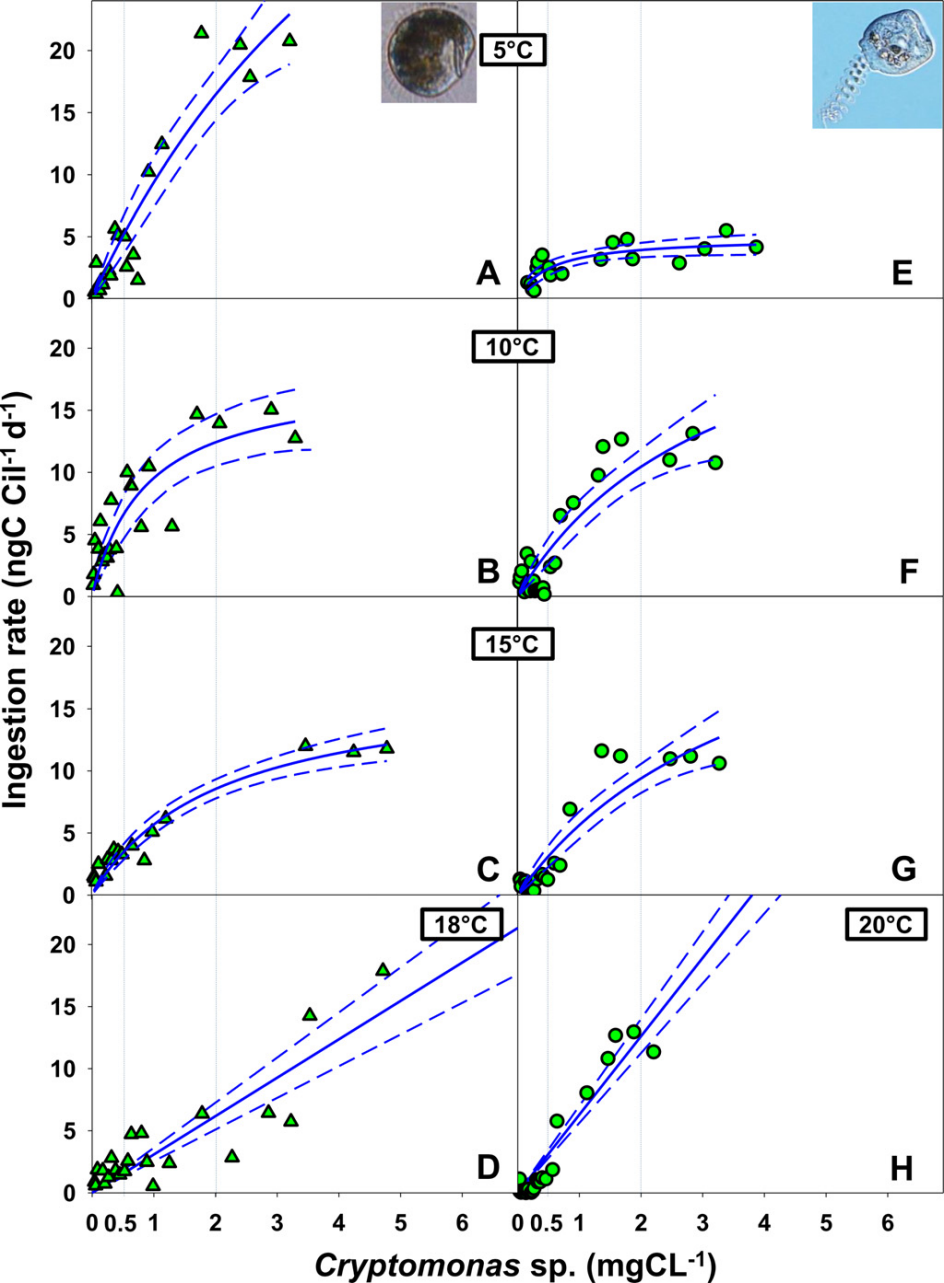
Functional response in Holling’s type II model of *Histiobalantium bodamicum* (**A–D**) and *Vorticella natans* (**E–H**) over temperature ranging from 5 to 20 °C and at food levels ranging from 0.02 to 7.0 mg C/liter. Vertical dotted lines show moderate (0.5 mg C/liter) and high (2.0 mg C/liter) food concentration. Dashed lines mark the 95% confidence band of the curves.

**Figure 5:**
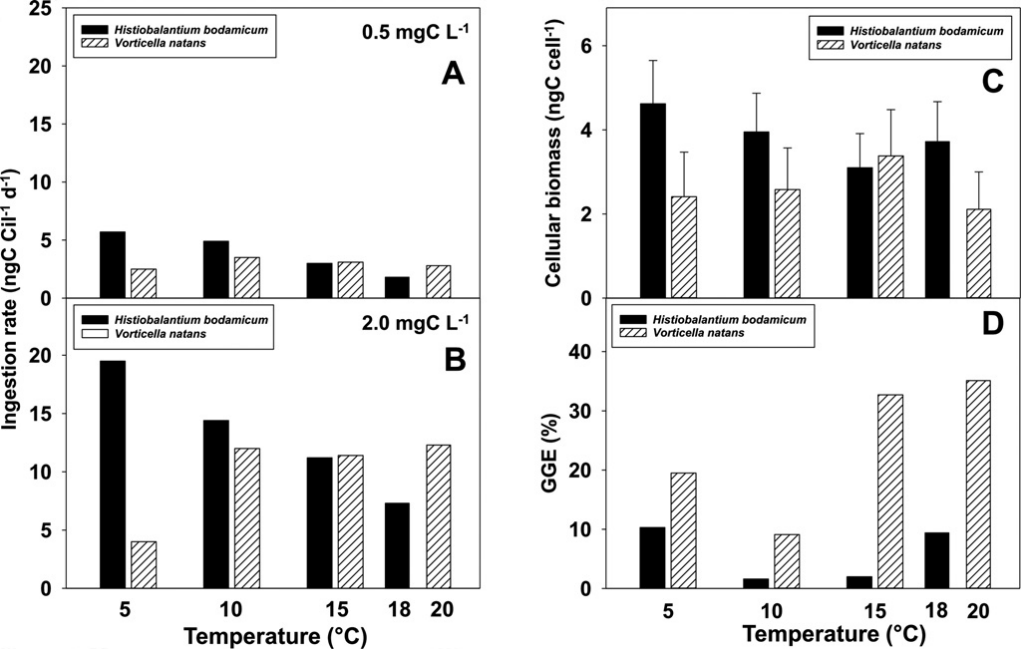
Temperature response from 5 to 20 °C of *Histiobalantium bodamicum* and *Vorticella natans*. (**A, B**) ingestion rate at 0.5 and 2.0 mg C/liter, respectively; (**C**) the relationship between the cellular biomass vs. temperature; (**D**) gross growth efficiency at 0.5 mg C/liter. Error bars in C represent standard errors.

We attempted to fit both species’ ingestion rates to Holling’s type II curvilinear response (Fig. 4A–C, E, F and Table 4). However, maximum ingestion rate (*I_max_*) was not reached at ecologically relevant food levels for both species, except for *V. natans* at 5 °C (Fig. 4E). At high temperatures, ingestion rates did not fit to Holling’s type II curvilinear response but showed a linear response (Fig. 4D, H). For arguments outlined above, we evaluated ingestion rates at moderate (0.5 mg C/liter) and high prey biomass (2.0 mg C/liter). Irrespective of food level, *I* of *H. bodamicum* decreased with increasing temperature (Fig. 5A, B and Table 4, 5). In *V. natans*, *I* appeared unaffected by temperature at moderate food level (Fig. 5A). At high prey biomass, ingestion rates of *V. natans* were significantly lower at 5 °C (4.0 mg C/liter) than at 10–20 °C (Fig. 5B), at which temperatures *I* was virtually constant (~12 mg C/liter).

**Table 4:** Parameter values (± SE) of functional responses for the curves presented in Fig. 4

Temperature (°C)		*I_max_* (ng C/Cil/d)	*p‐value*_*Imax*	*k* (ng C/Cil/d)	*p‐value*_*k*
5	*H. bodamicum*	67.35 ± 35.30	0.0725	6.22 ± 4.35	0.1705
	*V. natans*	4.94 ± 0.60	< 0.0001	0.52 ± 0.20	0.0179
10	*H. bodamicum*	17.81 ± 3.30	< 0.0001	0.87 ± 0.37	0.0302
	*V. natans*	27.68 ± 9.29	0.0064	3.31 ± 1.72	0.0659
15	*H. bodamicum*	17.37 ± 1.82	< 0.0001	2.07 ± 0.45	0.0003
	*V. natans*	28.54 ± 9.99	0.0085	4.09 ± 2.13	0.0660
18	*H. bodamicum*	infinite	1.0000	infinite	1.0000
20	*V. natans*	infinite	1.0000	infinite	1.0000

**Table 5:** Ingestion rates (*I*) and growth rates (*µ*) at prey levels of 0.5 mg C/liter and 2.0 mg C/liter

Temperature (°C)		*I_0.5_* (ng C/Cil/d)	*I_2.0_* (ng C/Cil/d)	*µ_0.5_* (d^−1^)	*µ_2.0_* (d^−1^)
5	*H. bodamicum*	4.8	16.3	0.13	0.20
	*V. natans*	2.4	4.0	0.22	0.30
10	*H. bodamicum*	6.3	12.0	0.02	0.26
	*V. natans*	3.9	10.7	0.13	0.34
15	*H. bodamicum*	3.2	8.3	0.02	0.24
	*V. natans*	2.8	9.2	0.31	0.41
18	*H. bodamicum*	1.5	6.3	0.19	0.36
20	*V. natans*	3.2	12.7	0.50	0.73

### Cellular biomass and gross growth efficiency in relation to temperature

Cellular biomass (Fig. 5C) and gross growth efficiency (Fig. 5D and Table 1) of *H. bodamicum* and *V. natans* were also affected by temperature. With one exception (at 15 °C), *H. bodamicum* had larger individual biomass than *V. natans*. From 5 to 15 °C, cellular biomass of *H. bodamicum* decreased from 4.5 to 3.0 ng C/cell (Fig. 5C), but was higher at 18 °C (3.6 ng C/cell). Cellular biomass of *V. natans* reached its maximum at 15 °C (3.3 ng C/cell) and was close to 2.3 ng C/cell at the other temperatures (Fig. 5C).

Gross growth efficiency (GGE) of *H. bodamicum* was low (≤ 10%) at moderate food level (0.5 mg C/liter) over the entire temperature range (Fig. 5D). In *V. natans*, GGE exceed 30% at 15–20 °C and was lowest (9.1%) at 10 °C. We did not calculate GGE at higher food levels, because cell volume of *Cryptomonas* sp. was only measured at ~ 0.5 mg C/liter (see Materials and Methods).

## Discussion

### Does feeding on bacteria affect ciliate growth and ingestion rates presented in this study?

We have ignored the potential effect of bacteria, which were always present in our experiments, on the results reported. This is mainly problematic for *V. natans* because, in contrast to *H. bodamicum*, *V. natans* is known as a voracious bacterial feeder, comparable to many sessile peritrich ciliates (Foissner et al. 1999). Bacterial uptake rates up to 19,000/ciliate/h have been measured for this species in hypertrophic fishponds at bacterial levels > 10^7^ cells/ml (Šimek et al. 2019). Lower rates, up to 1,100/ciliate/h, were estimated for *V. natans* from eutrophic Lake Oglethorpe, Georgia, USA (Sanders et al. 1989). However, the latter study used immobile fluorescent prey surrogates (microspheres), which are known to underestimate protist ingestion rates significantly (Bloem et al. 1988; Sherr et al. 1987). Including results from bacterivory experiments with other *Vorticella* species of comparable size (summarized by Weisse et al., in prep.), it seems likely that *V. natans* ingests several thousand bacteria/ciliate/h at bacterial densities of 10^6^–10^7^ cells/ml. Although we cannot rule out that such bacterial levels were reached in some of our experiments, their contribution to total potential prey biomass should have been minor. For instance, if we take the highest initial bacterial abundances in our experiments (i.e. 1.93 × 10^6^ cells/ml; Table S1) and assume that bacteria would have divided nearly three times in the course of our experiments, the geometric mean bacterial density calculated from Equation (2) would have been close to 5 × 10^6^ cells/ml, corresponding to ~ 0.53 mg C/liter. During the experiment, the highest initial *Cryptomonas* abundance increased on average by 13% to 113,000 cells/ml, corresponding to 3.8 mg C/liter, that is, it was 7.2‐fold higher than the estimated bacterial biomass. Only at the lowest *Cryptomonas* level, bacteria may have reached a biomass level similar to that of the algae.

To further examine the potential bacterial effect on ciliate growth rates, we estimated the bacterial biomass at each *Cryptomonas* level, assuming a linear relationship between bacterial biomass and *Cryptomonas* biomass using the data shown in Table S1 (Fig. S1). Then, we refitted the NR curves with the combined bacterial and *Cryptomonas* biomass (Fig. S2). The estimates of the threshold prey concentration *P'* increased slightly (0.14 mg C/liter vs. 0.10 mg C/liter), whereas the growth rate *µ* remained almost unchanged. We conclude that the bacterial effect on the NR curves was negligible in our study.

Similarly, to estimate to what extent we may have underestimated the total (i.e. *Cryptomonas* plus bacteria) ingestion rate of *V. natans* (*I*_max_, Table 4 and Fig. 4A–D), we can crudely calculate bacterial uptake rates from the available literature. Neglecting lower values reported by Sanders et al. (1989), estimates of maximum picoplankton clearance rates by *V. natans* are close to 0.7 µl/cil/h (Peštová et al. 2008; Šimek et al. 2019). These clearance rates were measured at water temperatures ranging mainly from 10 to 22 °C. Assuming a mean bacterial density of 2 × 10^6^ cells/ml in our experiments, *V. natans* may have taken up 1.4 × 10^3^ cells/cil/h, equivalent to 0.15 ng C/cil/h or 3.6 ng C/cil/d. Accordingly, the FR curves presented in Fig. 4 would shift up (Fig. S3), increasing *I*_max_ by about 20% at 10–22 °C. We did not calculate potential bacterial uptake rates at 5 °C because no data are available for bacterial ingestion rates of *V. natans* at temperatures < 10 °C.

Including bacterial uptake would reduce our estimates of gross growth efficiency (GGE) to 8% at 10 °C and 28–29% at 15 and 20 °C. Since we did not observe bacterial clusters in the food vacuoles of *V. natans* at the end of the experiments, we conclude that the above estimates represent maximum bacterial ingestion rates that were probably not reached in our study.

The foregoing considerations suggest that the motile species *V. natans* differs ecologically from many sessile peritrich ciliates, which are considered undiscriminative fine‐suspension feeders specialized to feed upon particles in the picoplankton size range (Fenchel 1987; Foissner et al. 1999). Our results challenge the conjecture that all *Vorticella* species are highly efficient picoplankton grazers (Šimek et al. 2019). Foissner et al. (1999) already documented algal cells in the food vacuoles of *V. natans*, similar to our observations (Fig. 1G–J). These authors also noted that planktonic peritrich species often feed upon small algae. More research is needed to investigate differences in the oral apparatus of motile vs. sessile peritrichs. In particular, experiments are required to test whether *V. natans* can discriminate between bacteria and small algae such as *Cryptomonas* spp.

### Functional ecology of *Vorticella natans* and *Histiobalantium bodamicum*—bottom‐up control

This is the first study investigating bottom‐up control of *V. natans* and *H. bodamicum* in relation to food and temperature. Our data demonstrate that both species are well adapted to cold conditions, tolerating 5 °C, while their UTTL differs.

The numerical and functional response data suggest that *V. natans* should be competitive in oligo‐mesotrophic lakes. Its growth and ingestion rates were comparable to those of other common planktonic ciliates under similar experimental conditions, for example *Rimostrombidium lacustris*, *Pelagostrombidium fallax*, *Balanion planctonicum*, and *Urotricha furcata* (Müller and Geller 1993; Müller and Schlegel 1999; Weisse et al. 2002). *Vorticella natans* has higher growth rates, higher affinity to prey (α), and lower threshold concentration (*P*′) than *H. bodamicum*. Gross growth efficiency of the latter was also lower (mostly < 10%) than that of *V. natans* (~10–35%) and most other planktonic ciliates (typically ~ 20–30%; reviewed by Straile 1997).

Sloppy feeding might explain why we did not observe food saturation in *H. bodamicum* at 18 °C (Fig. 4D). However, the unusually low GGE of *H. bodamicum* may also indicate that the prey organism, *Cryptomonas* sp., used in our study was suboptimal food for this ciliate. Müller and Schlegel (1999) had already studied NR and FR of a *H. bodamicum* strain isolated from Lake Constance with the same *Cryptomonas* sp. strain as food. These authors noted that *H. bodamicum* does not feed on nonmotile prey (small centric diatoms) and reported similar µ_max_ (0.33 ± 0.11 d^−1^) and lower *P*′ (0.29 mg C/liter) than the present study at 15 °C (µ_max_ = 0.38 ± 0.05 d^−1^, *P*′ = 0.46 ± 0.07 mg C/liter; Table 3). Similar to our study, Müller and Schlegel (1999) found that ingestion rates of *H. bodamicum* increased linearly over the entire range of prey concentrations (up to 1.5 mg C/liter) that they tested. Results of the present study indicate that food saturation of this peculiar ciliate at 18 °C was not even reached at 5 mg C/liter. Also, *H. bodamicum* was an inferior competitor relative to the choreotrich ciliate *Strobilidium lacustris* (syn. *Rimostrombidium lacustris*) and the prostome species *Balanion planctonicum* (Müller and Schlegel 1999).

Müller and Weisse (1994) compared growth rates of *H. bodamicum* measured in the laboratory to growth rates measured by enclosure experiments under *in situ* conditions. The in situ growth experiments conducted in L. Constance with the natural food (including bacteria) yielded positive results during autumn and winter, with µ_max_ = 0.40 d^−1^ at 17.2 °C. However, µ measured at this temperature was highly variable. Growth rates measured by Müller and Weisse (1994) in the laboratory at 18 °C were lower (0.25–0.33 d^−1^). Negative growth rates of *H. bodamicum* were repeatedly measured in L. Constance in summer when lake temperature exceeded 20 °C (Weisse 2006). We conclude that (i) the NR data provide realistic estimates of the temperature response of µ_max_ and *P*′ of this ciliate, (ii) the temperature optimum of this species is rather low (17–18 °C), and (iii) its growth rates rapidly decline above this temperature. Compared to *H. bodamicum*, *V. natans* tolerates higher water temperature (> 20 °C).

We reject our initial hypothesis (H_1_) because the previous studies and the present work suggest that, with respect to bottom‐up control, *V. natans* is the superior competitor, relative to *H. bodamicum*, in many temperate lakes. In seeming contrast to this inference, the natural abundance of *V. natans* is usually lower than that of *H. bodamicum*. For example, in Mondsee the average abundance recorded from June until November was 16‐fold higher for *H. bodamicum* (549 cells/liter) than for *V. natans* (33 cells/liter) (Kammerlander et al., pers. comm.). We assume that this is primarily due to top‐down control by microcrustaceans. Motility plays a key role in the susceptibility of ciliates to predation. *Vorticella natans* can be captured easily, because it moves slowly and seems to be insensitive to hydrodynamic disturbances generated by swimming predators (own unpubl. obs.). In contrast, *H. bodamicum* is highly sensitive to hydrodynamic disturbances, swims intermittently and fast, performing "jumps" that are typical of common freshwater ciliates to reduce the risk of being captured (Foissner et al. 1999; Weisse and Sonntag 2016). Top‐down experiments investigating predation pressure by common microcrustaceans on the two ciliates of the present study and some other planktonic ciliates are currently in progress in our laboratory. Preliminary results support differential susceptibility of *H. bodamicum* and *V. natans* to grazing by cladocerans and copepods. Details of the top‐down experiments will be reported elsewhere.

### Temperature response of planktonic protists—general implications

Our study confirmed earlier findings that virtually all ecophysiological parameters derived from NR and FR experiments are sensitive to temperature, with large species‐specific and intraspecific differences (Kimmance et al. 2006; Weisse 2004; Weisse et al. 2002; Yang et al. 2013). For instance, cell volume generally declines with temperature in planktonic protists (Atkinson et al. 2003; Montagnes and Franklin 2001; Montagnes et al. 2008). However, in ciliates an irregular response of cell volume to temperature has been reported for several species (Weisse 2004; Weisse et al. 2002). In our two study ciliates, the response of cellular biomass (calculated from cell volume, see Materials and Methods) to temperature was unimodal (Fig. 5C), but mirror‐inverted (i.e. peaking at 15 °C in *V. natans*, at which temperature *H. bodamicum* was smallest). The volume response of *V. natans* is similar to that obtained for the oligotrichine ciliate *Meseres corlissi* under comparable experimental conditions (Weisse 2004). Atkinson et al. (2003) reviewed several physiological causes of the inverse relationship between protist cell size and temperature but noted that no single explanation is widely accepted. It is undisputed that cell volume of protists is positively related to food supply (Fenchel 1987), and several studies reported significant temperature × food interaction for cell volume of ciliates and heterotrophic flagellates (Kimmance et al. 2006; Montagnes et al. 2008; Weisse et al. 2002). We suggest that the lack of an unequivocal response of cell volume to temperature in ciliates may result from its species specifically different interaction with resources (i.e. food).

Similar to cell volume, the temperature effect on the threshold food concentration (*P*′) was unimodal in both ciliate species, peaking at intermediate temperatures (Fig. 3E). This result is surprising because it suggests that the ciliates require higher food levels to sustain their populations at moderate temperatures than at lower and higher temperatures. In oligo‐mesotrophic Lake Mondsee, from which the ciliates were isolated, and similar temperate lakes phytoplankton maxima in spring and autumn are often reached at temperatures ranging from 10 to 15 °C (Bergkemper and Weisse 2018; Crosbie et al. 2003). However, it remains open if the reduced *P*′ at the temperature extremes, at which food levels are usually lower, is an adaptive response of the ciliates. An inverse temperature response of *P*′ had been observed with the same food organism for the prostome ciliate *Urotricha farcta* (Weisse et al. 2002), which is common in eutrophic water bodies (Foissner et al. 1999) and tolerant to a wide temperature range (Weisse et al. 2001).

The temperature effect on specific growth rates differed between the two ciliate species. In *H. bodamicum*, the increase of µ_max_ with temperature was linear, similar to most other planktonic ciliates studied thus far (Montagnes et al. 2003), but unusually low (0.23 ± 0.04 at 5 °C, 0.44 ± 0.06 at 18 °C; Fig. 3A and Table 3). The average linear temperature increase of µ_max_ obtained for ~ 20 ciliate species and other planktonic protists is 0.06–0.08 (d^–1^ °C^–1^) (Montagnes et al. 2003). In *V. natans*, µ calculated at 2.0 mg C/liter (Fig. 3C and Table 5) increased linearly between 5 and 15 °C at only 0.011 ± 0.002 (d^–1^ °C^–1^) and then increased over‐proportionately at 20 °C. Values recorded for *V. natans* at 20 °C were twofold to fourfold higher than measured at 10 °C, depending on food concentration (Fig. 3B, C and Table 5).

In agreement with previous empirical observations (Kimmance et al. 2006), the temperature effect on GGE was obvious in both ciliate species (Fig. 5D), supporting our second hypothesis (H_2_).

### A brief review on the taxonomic position of *Vorticella natans* based upon molecular markers and previous morphological and behavioral studies

*Vorticella natans* was originally described by Fauré‐Fremiet (1924). Jankowski (1985) transferred *V. natans* to the genus *Pelagovorticella* Jankowski, 1980, with *P. mayeri* as the type species, which was not accepted by Warren (1986), Foissner (1988), and Foissner et al. (1992). However, Foissner et al. (1999) reinstated the genus *Pelagovorticella*, based upon its planktonic life style, and reassigned *V. natans* as *Pelagovorticella natans*.

**Figure 6:**
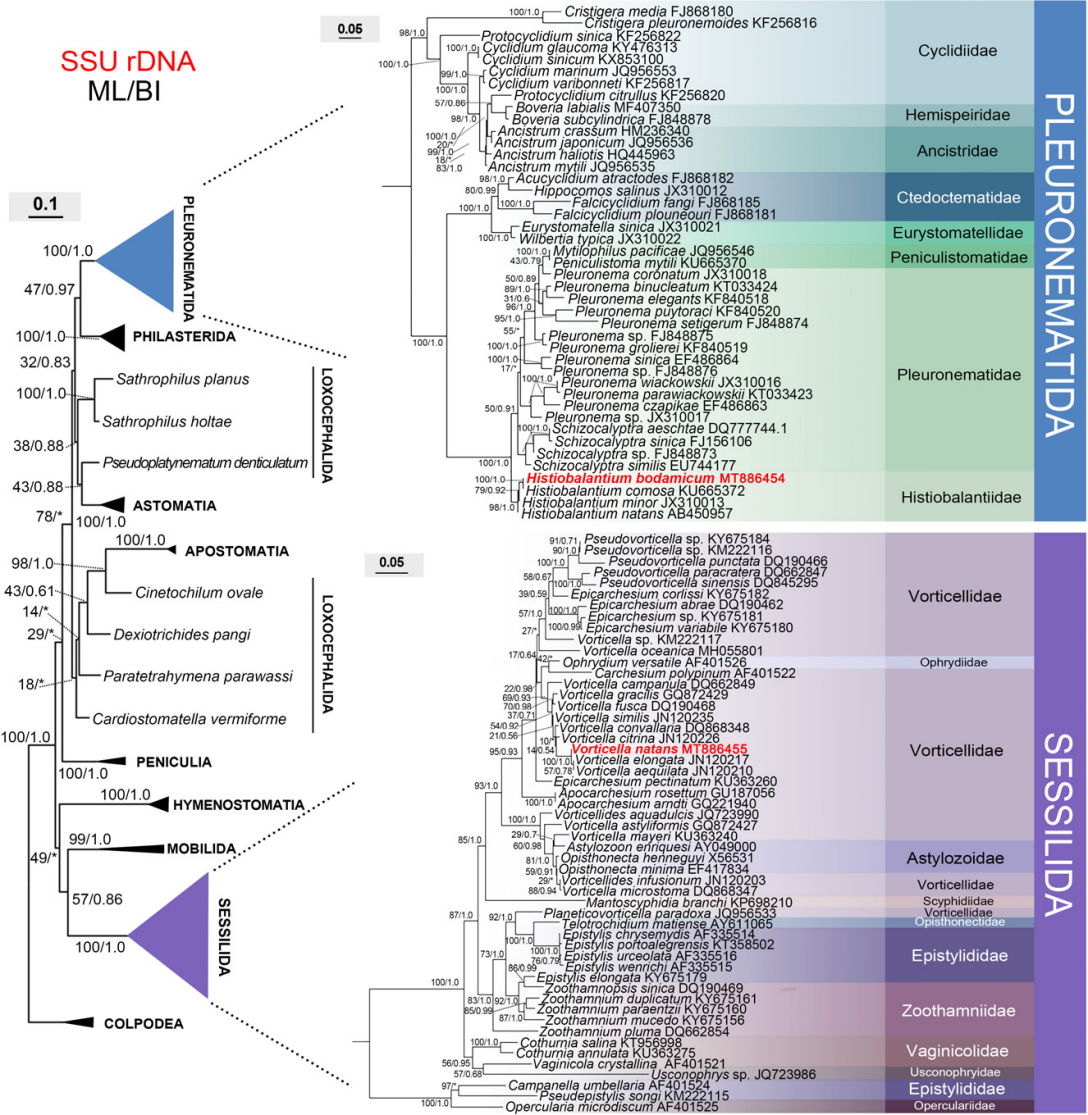
Maximum‐likelihood (ML) tree inferred from SSU rDNA sequences showing the systematic position of *Histiobalantium bodamicum* and *Vorticella natans* (bold). Numbers near nodes are nonparametric bootstrap values for ML and posterior probability values for Bayesian inference (BI). "*" refers to disagreement in topology with the BI tree. All branches are drawn to scale. The names of the species are updated, that is, do not always agree with those in GenBank. The scale bar corresponds to 10 and 5 substitutions per 100 nucleotide positions in the left tree and two right trees, respectively.

The length, GC content, and GenBank accession number of the SSU rDNA of *Histiobalantium bodamicum* were 1,646 bp, 42.22%, and MT886454, respectively. Length, GC content, and GenBank accession number of *Vorticella natans* were 1,603 bp, 42.79%, and MT886455. The phylogenetic trees of ML and BI shared a similar topology; therefore, only the ML tree is shown in Fig. 6 with the support values from both ML and BI algorithms. For clarity, we present both trees of Pleuronematida and Sessilida separately. In the tree of Pleuronematida, *H. bodamicum* branches within *Histiobalantiidae* with full support. In the tree of Sessilida, *V. natans* clusters with *Vorticella elongata* with full support. However, the type species of the (former) genus *Pelagovorticella* (*Pelago*‐), *Vorticella mayeri* clusters with the *Astylozoon enriquesi* clade with a very low bootstrap support value (29% ML, 0.70 BI). Based upon morphology and phylogenetic analyses of the SSU rDNA gene (this study, Fig. 6) and ITS1‐5.8S‐ITS2 sequences (Sun et al. 2016), we consider *Pelagovorticella* as a junior synonym and reassign this species to the genus *Vorticella*. The phylogenetic position of (*Pelago‐*) *Vorticella mayeri* awaits further research with more genetic markers.

## Conclusions

We characterized the functional ecology of two contrasting planktonic freshwater ciliates and inferred their competitive abilities with respect to bottom‐up control. We found similar maximum ingestion and growth rates at high food concentrations over temperatures ranging from 5 to 15 °C but pronounced differences between the species at higher temperatures. The lower food threshold, higher affinity to food at low levels, higher conversion efficiency, and wider temperature tolerance suggest that *V. natans* performs better than *H. bodamicum* in terms of bottom‐up control. The relatively low upper temperature tolerance of the latter species (~18 °C) may limit its occurrence in (the epilimnion of) temperate lakes if the ongoing lake warming continues. The unusually low GGE of *H. bodamicum* and its enigmatic FR recorded by Müller and Schlegel (1999) at 15 °C and in the present study at 18 °C require further testing with different food sources.

Temperature affected all parameters of the NR and FR curves of both species, but the temperature effect was species‐specific and did not always follow general trends derived from earlier studies and theoretical considerations. The underlying mechanisms driving ingestion and growth rates seem to respond differently to temperature (Weisse et al. 2016), explaining why the shape of the NR and FR curves do not change similarly with temperature. More research with other common ciliates is needed to better understand the nature of the temperature interaction in order to predict growth and ingestion rates of freshwater ciliates more accurately in situ.

## Supporting information


**Table S1.** Bacterial abundance and biomass in the experiments measured in three levels at 15 °C.
**Figure S1.** Estimated linear relationship between bacterial biomass and *Cryptomonas* biomass using the data shown in the Table S1.
**Figure S2.** Refitted numerical response (NR) curve with *Cryptomonas* and bacterial biomass combined (red dashed lines) and the original NR curves fitted with*Cryptomonas* biomass only (solid blue lines) of *Vorticella natans*.
**Figure S3.** Refitted functional response (FR) curves with *Cryptomonas* and bacterial biomass combined (red dashed lines) and the original FR curves fitted with*Cryptomonas* biomass only (solid blue lines) of *Vorticella natans*.
