# Supplementary material for: Functional Ecology of Two Contrasting Freshwater Ciliated Protists in Relation to Temperature
Source: J Eukaryot Microbiol. 2020 Dec 27;68(1):e12823. doi: 10.1111/jeu.12823 (PMC8044924; doi:10.1111/jeu.12823)
Supplement: Supplementary file 1 — Table S1. Bacterial abundance and biomass in the experiments measured in three levels at 15 °C. Figure S1. Estimated linear relationship between bacterial biomass and Cryptomonas biomass using the data shown in the Table S1. Figure S2. Refitted numerical response (NR) curve with Cryptomonas and bacterial biomass combined (red dashed lines) and the original NR curves fitted withCryptomonas biomass only (solid blue lines) of Vorticella natans. Figure S3. Refitted functional response (FR) curves with Cryptomonas and bacterial biomass combined (red dashed lines) and the original FR curves fitted withCryptomonas biomass only (solid blue lines) of Vorticella natans. [file JEU-68-0-s001.pdf]

SUPPORTING INFORMATION

Functional ecology of Two Contrasting Freshwater Ciliated Protists in Relation to Temperature
by Xiaoteng Lu, Yunyi Gao, Thomas Weisse

Table. S1. Bacterial abundance and biomass in the experiments measured in three levels at 15°C.

Fig. S1. Estimated linear relationship between bacterial biomass and *Cryptomonas* biomass using the data shown in the Supplementary Table S1.

Fig. S2. Refitted numerical response (NR) curve with *Cryptomonas* and bacterial biomass combined (red dashed lines) and the original NR curves fitted with *Cryptomonas* biomass only (solid blue lines) of *Vorticella natans*. Red triangle symbols and blue spherical symbols mark the refitted and original plots, respectively.

Fig. S3. Refitted functional response (FR) curves with *Cryptomonas* and bacterial biomass combined (red dashed lines) and the original FR curves fitted with *Cryptomonas* biomass only (solid blue lines) of *Vorticella natans*. Red triangle symbols and blue spherical symbols mark the refitted and original plots, respectively.

Table. S1. Bacterial abundance and biomass in the experiments measured in three levels at 15°C.

Cryptomonas conc. (cells mL^{-1})	*Cryptomonas* carbon (mgC L^{-1})	Bacteria conc. (cells mL^{-1})	Bacteria carbon (mgC L^{-1})	*Cryptomonas* vs. Bacteria carbon ratio
1000	0.03	323875	0.035	**1:1**
15000	0.51	608886	0.065	**8:1**
100000	3.39	1930298	0.206	**16:1**

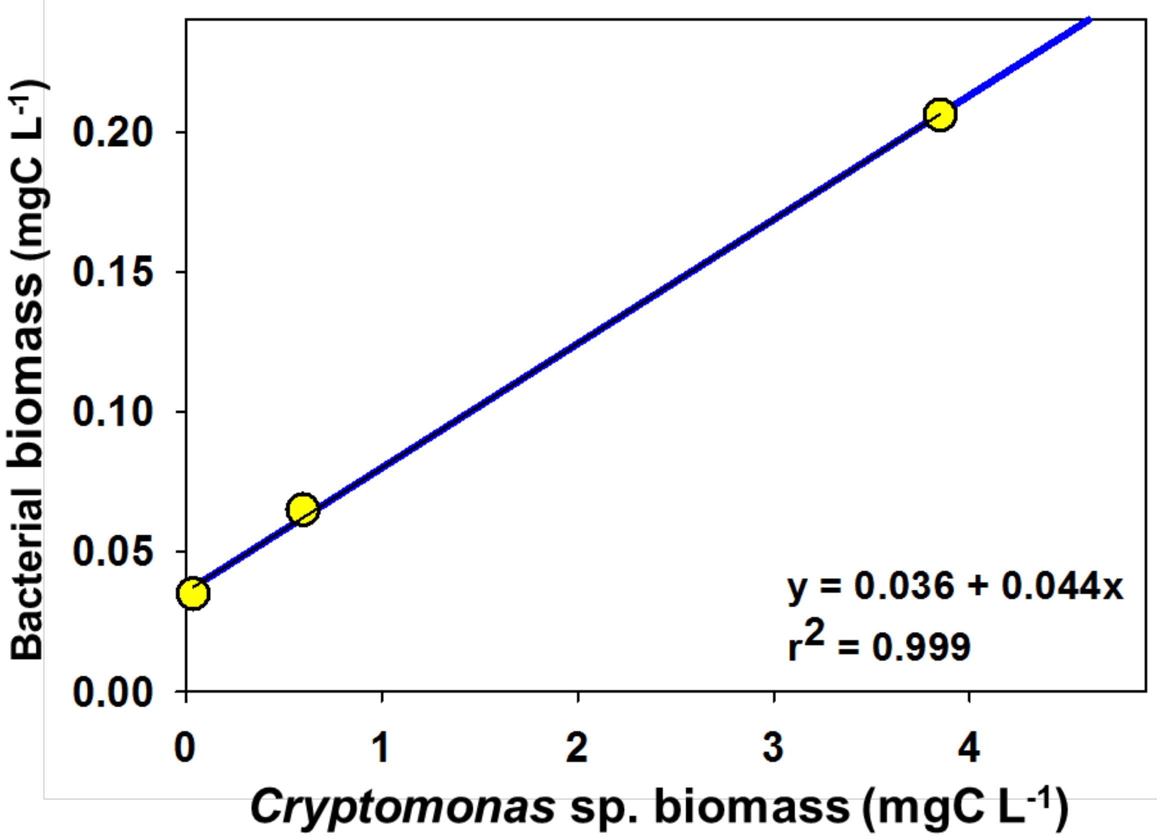

Bacterial biomass (mgC L-1)
0.20
0.15
0.10
0.05
0.00
0
1
2
3
4
Cryptomonas sp. biomass (mgC L-1)
y = 0.036 + 0.044x
r2 = 0.999

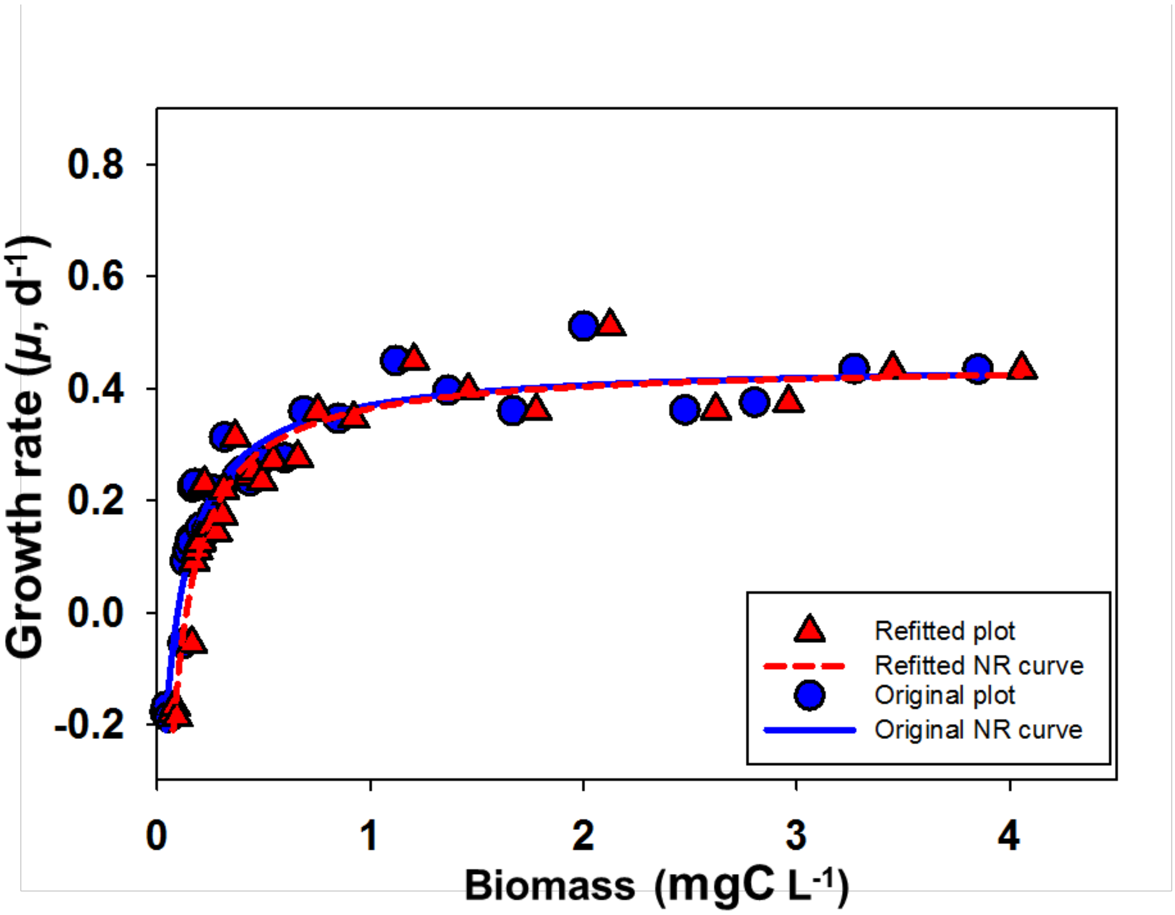

Growth rate (μ, d-1)
0.8
0.6
0.4
0.2
0.0
-0.2
0
1
2
3
4
Biomass (mgC L-1)
Refitted plot
Refitted NR curve
Original plot
Original NR curve

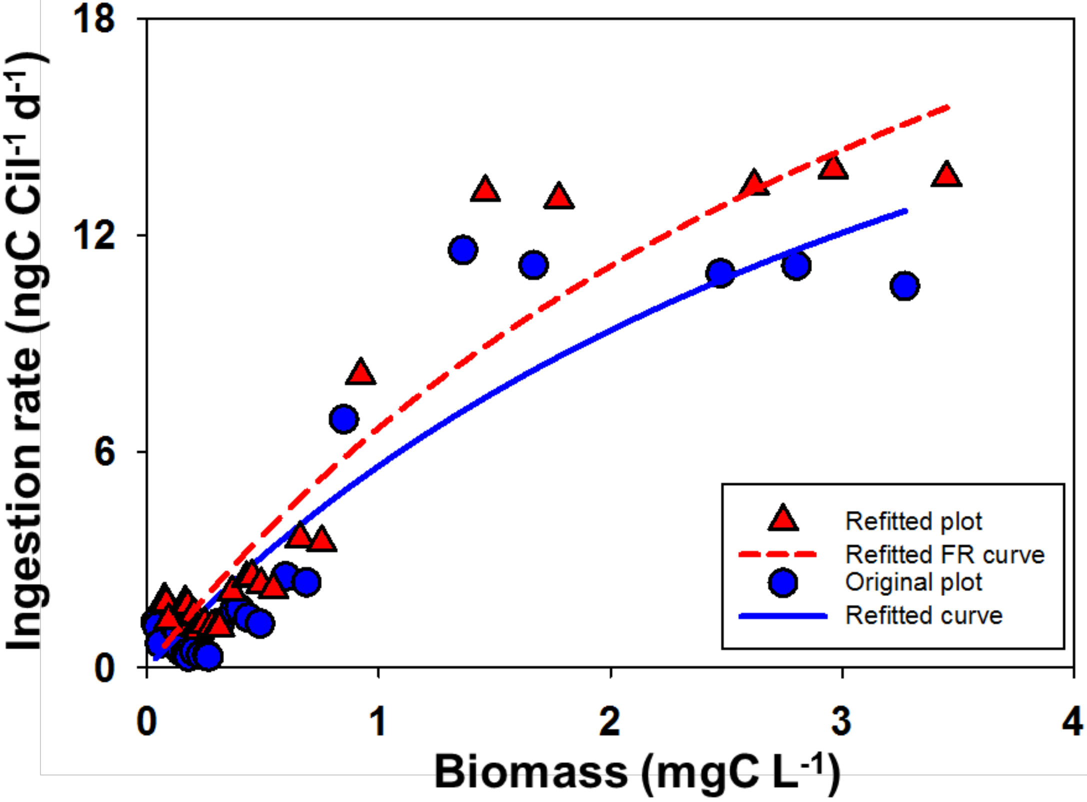

Ingestion rate (ngC Cil-1 d-1)
18
12
6
0
0
1
2
3
4
Biomass (mgC L-1)
Refitted plot
Refitted FR curve
Original plot
Refitted curve
